# Interactive interfaces and wearable technologies for enhancing health management among older adults: a systematic review

**DOI:** 10.3389/fmed.2025.1724828

**Published:** 2025-12-15

**Authors:** Chenghong Cen, Hairong Peng, Xinru Li, Zhuoxian Zhang, Jiaqi Hu, Zikai Wang, Tan Jiang

**Affiliations:** 1 School of Fine Arts, South China Normal University, Guangzhou, China; 2 College of Publishing, University of Shanghai for Science and Technology, Shanghai, China; 3 School of Games and Creative Technology, University for the Creative Arts, Farnham, United Kingdom

**Keywords:** health management, healthy aging, interactive interfaces, older adults, wearable technologies

## Abstract

**Background:**

Population aging has intensified the need for innovative approaches to support health and well-being among older adults. Interactive interfaces and wearable technologies have been proposed as promising tools for enhancing engagement in digital health management, yet evidence regarding their effectiveness and adoption remains inconsistent.

**Objective:**

This study aimed to systematically review and synthesize current evidence on the types of interactive interfaces and wearable technologies used with older adults, their impact on health engagement and management, and the barriers affecting adoption.

**Methods:**

This study conducted a mixed-methods systematic review to synthesize empirical evidence on interactive interfaces and wearable technologies for older adults. Two reviewers (A and B) independently screened and appraised all records, with a third reviewer (C) resolving disagreements. Database searches were conducted between January 2021 and August 2025 across PubMed, Scopus, Web of Science and CINAHL. A total of 1,474 records were identified, of which 101 full-text articles were assessed, and 48 studies met inclusion criteria. The review followed the Preferred Reporting Items for Systematic Reviews and Meta-Analyses (PRISMA) guidelines and employed the Mixed Methods Appraisal Tool (MMAT) for quality assessment. Thematic analysis was applied to extract and integrate findings across diverse study designs.

**Results:**

Five categories of technologies were identified: commercial wearable devices, custom-built systems, mobile health platforms, age-friendly interface designs, and immersive or alternative modalities. These technologies influenced health engagement through four main pathways: usability and ease of learning, self-monitoring and adherence, motivational support, and psychosocial empowerment. Nevertheless, multiple barriers constrained adoption, including usability limitations, low digital literacy, privacy and trust concerns, and financial and sustainability challenges. Quality assessment revealed considerable methodological variability, with only a subset of studies achieving high rigor.

**Conclusion:**

Overall, the findings demonstrate that while interactive interfaces and wearable technologies can empower older adults in managing their health, their broader impact depends on user-centered design, training and support mechanisms, transparent data practices, and equitable access models. The review provides an integrative framework that connects interface and wearable design to health management outcomes, offering practical guidance for inclusive and sustainable digital-health innovation for aging populations.

## 1 Introduction

Population aging is one of the most significant demographic shifts confronting societies worldwide (1). The United Nations defines population aging as the increasing proportion of older persons within a population, driven by declining fertility rates and increasing life expectancy. Aging refers to the progressive biological, functional, and psychosocial changes that occur over time, influencing health needs, functional capacity, and patterns of technology use. According to the World Health Organization, older adults are commonly defined as individuals aged 60 years and above, reflecting both global demographic conventions and gerontological practice. As the proportion of older adults rises, the burden of managing chronic conditions and maintaining functional independence increases accordingly. Traditional health care models are often not sufficient to meet the complex needs of aging populations, which has motivated growing interest in digital health technologies as supportive tools (2). In this context, interactive interfaces and wearable technologies have emerged as promising avenues for enhancing older adults’ engagement in health management particularly through capabilities like real-time monitoring, feedback loops, and self-management assistance (3–5).

However, adopting and sustaining use of these technologies is nontrivial for older populations, which face distinct physical, cognitive, usability, and motivational challenges (6). Age-related declines in vision, motor control, and cognition may reduce adoption and compromise the usability of standard interfaces, including small fonts and complex menus (7). Moreover, older users often balance perceived benefits against concerns related to privacy, cost, trust, and learning burden (8).

While many reviews have addressed either wearable technologies or user interface design separately, fewer have systematically synthesized how interactive interface design and wearable technologies jointly influence health engagement and management in older adults. Yet, growing empirical evidence indicates that these two domains are inherently interdependent rather than distinct. Wearable devices rely on interface design to transform sensor data into meaningful feedback, comprehension, and motivation, while interface usability directly mediates older adults’ interpretation of data and adherence to health behaviors (9–11). In other words, the effectiveness of wearables is inseparable from the quality of their interface design, which shapes users’ perceived control, trust, and emotional engagement. This data–interaction loop reflects a socio-technical coupling between sensing and interaction that determines whether technology can support sustained engagement.

For example, a recent scoping review on digital engagement among older adults emphasized that engagement involves not only access and usability, but also sustained motivation, emotional responses, and adaptability over time (8). Another review of mobile health (mHealth) apps tailored for older adults highlighted design recommendations such as large buttons and simple navigation, but did not deeply explore the role of wearable devices in reinforcing engagement and health management behaviors (12). By jointly examining wearable technologies and interface design, this review aims to capture the full interaction process: how sensory feedback, data visualization, and user interpretation coalesce to produce engagement outcomes in aging populations.

Furthermore, wearable devices have demonstrated promising potential: for instance, they allow continuous physiological monitoring, fall detection, and activity tracking in real-world settings, which can support older adults’ autonomy and preventive care (11, 13). Yet, uptake is uneven. Many studies report usability, comfort, data reliability, and cost barriers that limit sustained use in older populations (14).

Given these gaps, there is a need for a systematic review that addresses the intersection of interface design and wearable technology in older adult populations, focusing specifically on how they facilitate digital health engagement and management. To clarify this conceptual intersection, this review introduces an analytical perspective termed the Interface–Wearable Engagement Pathways (I-WEP) framework, which views interactive interfaces and wearable technologies as elements of a single socio-technical system. Within this system, sensor-based data, interface feedback, and user actions form iterative feedback loops that shape digital health engagement and management. The I-WEP framework delineates four primary pathways through which these technologies exert influence: (P1) usability and learnability, (P2) self-monitoring and adherence, (P3) motivational supports, and (P4) psychosocial empowerment. This framework provides an integrated lens for synthesizing heterogeneous findings and for explaining how design and interaction mediate behavioral and experiential outcomes in older adults.

More concretely, this review is guided by the following research questions:

- RQ1: What types and design features of interactive interfaces and wearable technologies have been deployed with older adults in digital health contexts?
- RQ2: To what extent do these modalities influence older adults’ digital health engagement and management?
- RQ3: What are the principal barriers, limitations, and research gaps in current studies on these technologies with older adult populations?

By synthesizing the evidence, we aim to provide design and research recommendations for future development of more effective and inclusive digital health solutions for the aging population.

## 2 Methods

### 2.1 Search strategy

This review was conducted as a mixed-methods systematic review, integrating both quantitative and qualitative evidence on interactive interfaces and wearable technologies for older adults. The methodological framework followed the Preferred Reporting Items for Systematic Reviews and Meta-Analyses (PRISMA) 2020 guidelines and applied a thematic synthesis approach, drawing upon the principles proposed by Thomas and Harden in 2008 (15) for qualitative integration. The Mixed Methods Appraisal Tool (MMAT, version 2018) was used to assess the methodological quality of included studies, thereby allowing for the inclusion of diverse designs within a coherent evaluative framework. This approach ensured consistency, transparency, and rigor in synthesizing heterogeneous forms of evidence across study types.

A comprehensive literature search was designed to identify empirical studies examining the role of interactive interfaces and wearable technologies in supporting digital health engagement and management among older adults. Four electronic databases (PubMed, Scopus, Web of Science, and CINAHL) were systematically searched. The initial search was conducted in July 2025, with an update performed in September 2025 to ensure inclusion of the most recent publications available up to August 2025. The search covered studies published between January 2021 and August 2025, and combined controlled vocabulary terms with free-text keywords. The strategy integrated three conceptual domains: (a) the target population (e.g., “older adults,” “elderly,” “seniors”), (b) the technological intervention (e.g., “interactive interface,” “wearable device,” “smartwatch,” “fitness tracker”), and (c) the outcomes of interest (e.g., “health management,” “self-management,” “health”). Boolean operators (AND, OR) and truncation were applied to maximize both sensitivity and specificity. The complete set of search terms and combinations used for each database is presented in Table 1. In addition, the reference lists of all included articles and relevant reviews were screened to capture further eligible studies.

**Table 1:** Keywords used for the search strategy.

Keywords
(“older adults” OR elderly OR senior OR “aging population” OR “aged 60 plus”) AND (“interactive interface” OR “user interface” OR “human-computer interaction” OR “wearable device” OR smartwatch OR “fitness tracker” OR “sensor technology” OR “mHealth device”) AND (“health management,” “self-management,” “health”)

In addition to database searches, backward and forward citation tracking was conducted for all included articles to ensure completeness. Grey literature, such as dissertations, institutional reports, and non-peer-reviewed sources, was excluded to preserve methodological rigor and ensure that all included evidence met peer-review quality standards. Only peer-reviewed studies published in English were included. While the availability of automated translation tools has improved, inclusion was limited to English-language publications to ensure accurate interpretation of methodological details, minimize misclassification risk during thematic synthesis, and maintain consistency with prior systematic reviews in digital health. Prior to the full search, a pilot test was conducted in PubMed and Scopus using a subset of search terms to ensure adequate coverage and precision of the retrieved records. The search string and inclusion criteria were refined iteratively based on this pilot to optimize sensitivity and specificity across databases.

### 2.2 Eligibility criteria

Studies were selected based on predefined eligibility criteria to ensure relevance and rigor. The population of interest included older adults aged 60 years and above, or studies explicitly targeting this demographic. For conceptual clarity, this review adopted standardized definitions of the key technological domains. According to the International Organization for Standardization (ISO 9241-110:2020), an interactive interface refers to “a user-facing digital system enabling bidirectional human–computer communication through input or output modalities such as touch, gesture, or voice.” In digital health contexts, this term encompasses applications, dashboards, or embedded user interfaces that mediate interpretation, feedback, and behavioral engagement (16). Consistent with Piwek et al. (17), wearable technologies are defined as sensor-embedded, body-worn devices, such as smartwatches, wristbands, or textile sensors, that continuously or intermittently capture physiological or behavioral data to support monitoring, feedback, or self-management. Although all wearables contain embedded software, classification in this review was based on the dominant mode of user interaction: studies focusing on sensing accuracy or ergonomics were categorized as wearable-focused, while those emphasizing usability, visualization, or engagement mechanisms were categorized as interface-focused. Interactive interfaces included mobile dashboards, touch- or gesture-based systems, voice assistants, and immersive or gamified health applications, but excluded purely recreational video games unrelated to health or well-being.

Eligible interventions therefore comprised interactive interfaces and wearable technologies designed to support digital health engagement and management among older adults. Studies were included if they reported empirical findings on outcomes related to health engagement, usability, acceptability, health behaviors, or other indicators of health management among older adults. Both qualitative and quantitative study designs, as well as mixed-methods studies, were considered, provided they were peer-reviewed and published in English.

Exclusion criteria removed studies that did not focus on older adults or failed to disaggregate data for this population; interventions not involving interactive interfaces or wearable devices; purely technical papers without user evaluation; and studies lacking health-related outcomes. Reviews, editorials, conference abstracts, and non-peer-reviewed publications were also excluded (Table 2).

**Table 2:** Inclusion and exclusion criteria.

Inclusion criteria	
Population	Studies involving older adults (typically aged 60 years or above) or populations explicitly described as elderly/seniors.
Intervention/Technology	Use of interactive interfaces, wearable devices, mobile health (mHealth) applications, or other digital tools that involve direct user interaction and aim to enhance engagement, self-management, or health-related behaviors among older adults.
Outcomes	Studies reporting on digital health engagement, health management, self-management, adherence, usability, user acceptance, or related outcomes.
Study design	Randomized controlled trials (RCTs), quasi-experimental studies, observational studies, qualitative studies, or mixed-methods studies.
Setting	Any setting where the intervention occurs, including home, community, clinical, or virtual environments.
Language	Studies published in English.
Publication date	Studies published within a defined timeframe (e.g., 2021–2025) to ensure relevance to current technologies.

Exclusion criteria	
Population	Studies focusing exclusively on children, adolescents, or non-elderly adults.
Intervention/Technology	Interventions not involving digital tools or interactive technologies (e.g., traditional health education programs, paper-based interventions); Digital tools that do not require user interaction or do not aim to promote engagement or health management, such as passive data collection platforms, back-end analytics, or electronic health record systems without patient-facing components.
Outcomes	Studies not reporting outcomes related to engagement, self-management, health management, or user interaction.
Study design	Editorials, commentaries, opinion pieces, conference abstracts without full data, protocols, or review articles.
Setting	Studies focusing solely on theoretical models or simulations without real-world application, unless directly relevant to user engagement or health management
Language	Studies published in non-English.
Publication Date	Publication date: Studies published outside the defined timeframe (e.g., before 2021).

### 2.3 Quality assessment

The methodological quality of the included studies was evaluated using the Mixed Methods Appraisal Tool (MMAT), version 2018, which is designed to appraise qualitative, quantitative, and mixed-methods research within a single framework. The MMAT includes two initial screening questions applicable to all studies, followed by five methodological criteria tailored to each study design. Each criterion was rated as Yes, No, or Cannot tell. Two reviewers (A and B) independently assessed all included studies, and any discrepancies were resolved through discussion or consultation with a third reviewer (C). The results of the quality appraisal are presented in a summary Table 3, indicating the extent to which each study met the MMAT criteria. This process allows for a transparent evaluation of methodological rigor and helps to contextualize the findings of the review.

**Table 3:** The result of MMAT evaluation.

Study	Screening questions (for all types)		Study design	Q1	Q2	Q3	Q4	Q5	Overall appraisal
	S1	S2							
Wang et al., 2025 (18)	Yes	Yes	Quantitative descriptive	Cannot tell	Cannot tell	Yes	Cannot tell	Cannot tell	1/5
Ma et al., 2023 (19)	Yes	Yes	Mixed methods	Yes	Cannot tell	Cannot tell	Cannot tell	Yes	2/5
Shim et al., 2024 (39)	Yes	Yes	Quantitative descriptive	YES	Cannot tell	Yes	Cannot tell	Yes	3/5
Joymangul et al., 2024 (64)	Yes	Yes	Quantitative descriptive	Cannot tell	Cannot tell	Yes	Cannot tell	Cannot tell	1/5
Ghosh et al., 2023 (35)	Yes	Yes	Quantitative descriptive	Cannot tell	Cannot tell	Yes	Cannot tell	Cannot tell	1/5
Mathew et al., 2024 (40)	Yes	Yes	Quantitative randomized controlled trials	Cannot tell	Cannot tell	Cannot tell	Cannot tell	Yes	1/5
Hsu et al., 2023 (20)	Yes	Yes	Quantitative descriptive	Cannot tell	Cannot tell	Cannot tell	Cannot tell	Yes	1/5
Schmidle et al., 2022 (58)	Yes	Yes	Quantitative descriptive	Cannot tell	Cannot tell	Yes	Cannot tell	Yes	2/5
Um et al., 2025 (31)	Yes	Yes	Quantitative descriptive	Cannot tell	Cannot tell	Yes	Cannot tell	Yes	2/5
Alnanih et al., 2023 (38)	Yes	Yes	Mixed methods	YES	Cannot tell	Cannot tell	Cannot tell	Cannot tell	1/5
Choma et al., 2023 (41)	Yes	Yes	Quantitative descriptive	Cannot tell	Yes	Yes	Cannot tell	Yes	3/5
Lee et al. 2025 (42)	Yes	Yes	Quantitative descriptive	Cannot tell	Cannot tell	Cannot tell	Cannot tell	Cannot tell	0/5
Ocagli et al., 2021 (56)	Yes	Yes	Quantitative descriptive	Cannot tell	Cannot tell	Yes	Cannot tell	Cannot tell	1/5
Kari et al., 2023 (59)	Yes	Yes	Quantitative descriptive	Cannot tell	Cannot tell	Yes	Cannot tell	Yes	2/5
Kastelic et al., 2021 (63)	Yes	Yes	Quantitative descriptive	Cannot tell	Cannot tell	Yes	Cannot tell	Yes	2/5
Alpert et al., 2024 (43)	Yes	Yes	Qualitative	Yes	YES	Yes	Yes	Yes	5/5
Liu et al., 2025 (21)	Yes	Yes	Quantitative descriptive	Yes	Cannot tell	Yes	Cannot tell	Cannot tell	2/5
Zhang et al., 2025 (22)	Yes	Yes	Quantitative descriptive	Yes	Cannot tell	Yes	Cannot tell	Yes	3/5
Liang et al., 2025 (23)	Yes	Yes	Quantitative descriptive	Yes	Cannot tell	Yes	Cannot tell	YES	3/5
Abouzahra et al., 2022 (44)	Yes	Yes	Qualitative	Yes	YES	Yes	Yes	Yes	5/5
Velciu et al., 2023 (65)	Yes	Yes	Quantitative descriptive	Yes	Cannot tell	Yes	Cannot tell	Cannot tell	2/5
Allah et al., 2023 (37)	Yes	Yes	Quantitative non-randomized	Yes	Cannot tell	Yes	Cannot tell	Yes	3/5
Villalobos et al., 2022 (45)	Yes	Yes	Quantitative descriptive	Yes	Cannot tell	Yes	Cannot tell	Yes	3/5
Ma et al., 2024 (24)	Yes	Yes	Quantitative non-randomized	Yes	Yes	Yes	Cannot tell	Yes	4/5
Arnaert et al., 2023 (54)	Yes	Yes	Qualitative	Yes	Yes	Yes	Yes	Yes	5/5
Paramasivam et al., 2024 (36)	Yes	Yes	Quantitative descriptive	Yes	Cannot tell	Yes	Cannot tell	Yes	3/5
Mardini et al., 2021 (46)	Yes	Yes	Quantitative descriptive	Yes	Cannot tell	Yes	Cannot tell	Yes	3/5
Wu et al., 2021 (47)	Yes	Yes	Mixed methods	Yes	Cannot tell	Cannot tell	Cannot tell	Cannot tell	1/5
Batsis et al., 2021 (60)	Yes	Yes	Quantitative descriptive	Cannot tell	Cannot tell	Yes	Cannot tell	Yes	2/5
Cotter et al., 2023 (48)	Yes	Yes	Quantitative randomized controlled trials	YES	Yes	Yes	Cannot tell	Cannot tell	3/5
Chung et al., 2022 (49)	Yes	Yes	Mixed methods	Yes	Yes	Yes	No	Yes	4/5
Song et al., 2024 (32)	Yes	Yes	Quantitative non-randomized	Cannot tell	Yes	Cannot tell	Yes	Cannot tell	2/5
Lin et al., 2023 (25)	Yes	Yes	Quantitative descriptive	YES	No	Yes	Yes	No	3/5
Seok et al., 2022 (33)	Yes	Yes	Quantitative non-randomized	Cannot tell	Yes	Cannot tell	Cannot tell	Cannot tell	1/5
Nascimento et al., 2025 (55)	Yes	Yes	Quantitative non-randomized	Cannot tell	Yes	Cannot tell	Cannot tell	Cannot tell	1/5
Wu et al., 2025 (26)	Yes	Yes	Quantitative non-randomized	Cannot tell	Yes	Cannot tell	Yes	Yes	3/5
Lin et al., 2024 (27)	Yes	Yes	Qualitative	Cannot tell	Yes	Yes	Yes	Yes	4/5
Flynn et al., 2022 (61)	Yes	Yes	Qualitative	Yes	Yes	Yes	Yes	Yes	5/5
Orlofsky et al., 2022 (50)	Yes	Yes	Qualitative	Yes	Yes	Yes	Yes	Yes	5/5
Tanaka et al., 2024 (34)	Yes	Yes	Qualitative	Yes	Yes	Yes	Yes	Yes	5/5
Silveira et al., 2020 (51)	Yes	Yes	Qualitative	Cannot tell	Yes	Yes	Yes	Yes	4/5
Martinato et al., 2021 (57)	Yes	Yes	Quantitative descriptive	Cannot tell	Cannot tell	Yes	Cannot tell	Yes	2/5
Wang et al., 2025 (28)	Yes	Yes	Quantitative randomized controlled trials	Yes	Yes	Cannot tell	Cannot tell	Cannot tell	2/5
Low et al., 2024 (52)	Yes	Yes	Quantitative descriptive	Yes	Cannot tell	Yes	Cannot tell	Yes	3/5
Wong et al., 2024 (29)	Yes	Yes	Qualitative	Yes	Yes	Yes	Yes	Yes	5/5
Jiang et al., 2023 (53)	Yes	Yes	Quantitative descriptive	Yes	Yes	Yes	Cannot tell	Yes	4/5
Lee et al., 2021 (30)	Yes	Yes	Quantitative non-randomized	Cannot tell	Yes	Cannot tell	Cannot tell	Cannot tell	1/5
Neves et al., 2023 (62)	Yes	Yes	Quantitative randomized controlled trials	Cannot tell	Cannot tell	Cannot tell	Yes	Yes	2/5

### 2.4 Data selection

**Figure 1:**
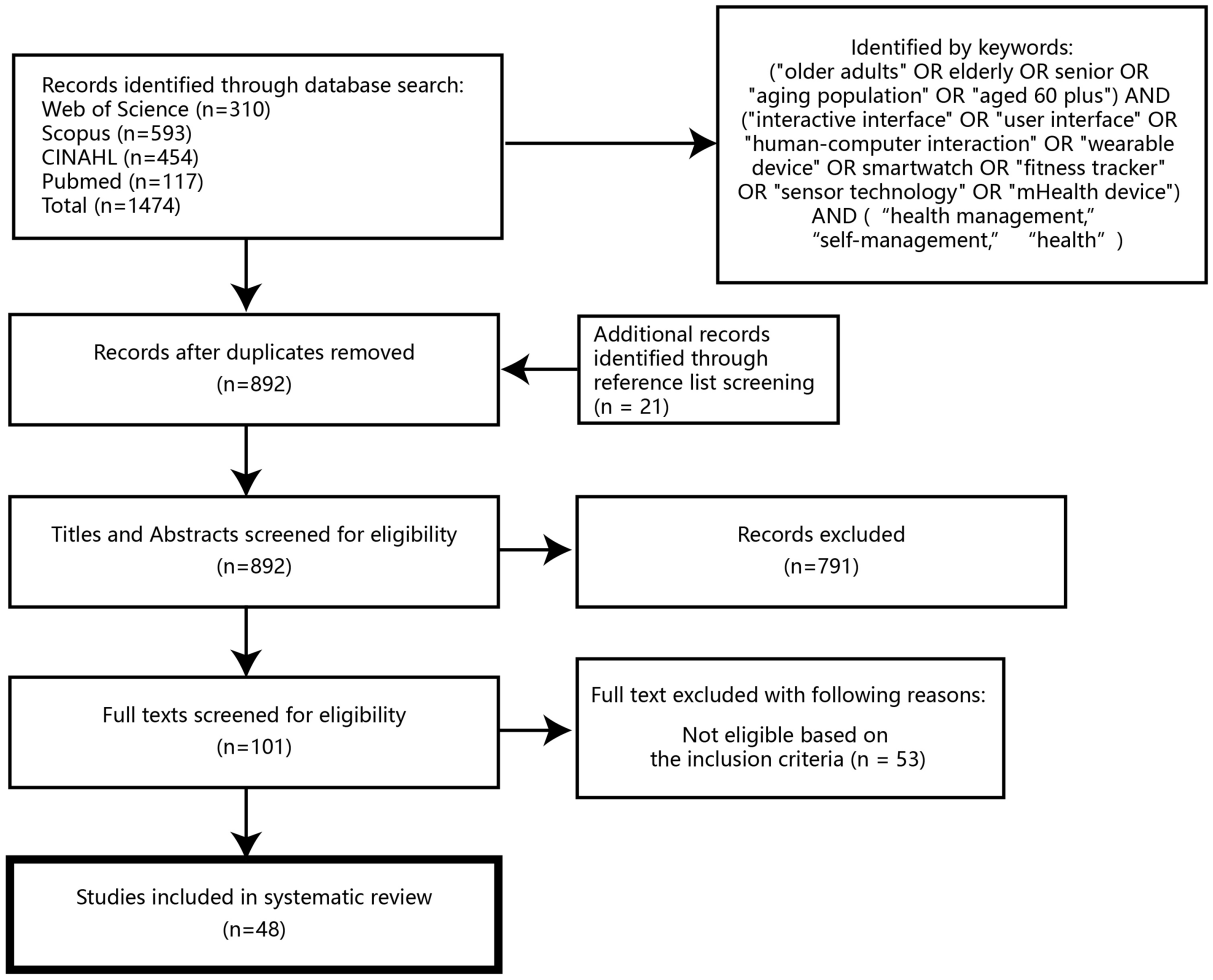
PRISMA flow diagram.

All records retrieved from the database searches were imported into EndNote reference management software, and duplicate entries were removed. The selection process was conducted in two stages. First, titles and abstracts were screened to exclude studies that clearly did not meet the eligibility criteria. Second, the full texts of potentially relevant studies were reviewed to determine their inclusion. Both stages were carried out independently by two reviewers (A and B), and any disagreements were resolved through discussion or, if necessary, adjudication by a third reviewer (C). Reasons for exclusion at the full-text stage were documented to ensure transparency. The entire selection process is reported in a PRISMA 2020 flow diagram (Figure 1), which summarizes the number of records identified (*n* = 1,474), screened (*n* = 892), excluded (*n* = 844), and included in the final synthesis (*n* = 48). In addition, the reference lists of all included studies and relevant reviews were manually screened to identify any additional eligible records, which were incorporated into the PRISMA flow as supplementary sources.

### 2.5 Data extraction and thematic analysis

A structured extraction form was developed based on PRISMA 2020 recommendations. The form included the following fields: author and year, study design, intervention type, and key findings related to engagement and health management. These fields were subsequently summarized in the synthesized tables. Data extraction was conducted independently by two reviewers (A and B). Any discrepancies were first discussed between the two reviewers and, if unresolved, were adjudicated by a third reviewer (C) to ensure accuracy and methodological rigor.

The extracted data were synthesized using a thematic analysis approach. This process involved several iterative steps. First, the reviewers familiarized themselves with the findings of the included studies by reading and re-reading the extracted data. Second, open coding was applied to identify salient concepts directly relevant to the research questions, such as usability, accessibility, self-monitoring, digital literacy, motivation, or barriers to adoption. Third, codes were organized into broader themes and subthemes that capture patterns across the studies. Themes were reviewed, refined, and defined to ensure conceptual coherence and distinction. Finally, the results were presented in a narrative synthesis, supported by thematic maps to illustrate the relationships between themes. This approach allowed for the integration of diverse forms of evidence while highlighting both facilitators and challenges in the use of interactive interfaces and wearable technologies for digital health management and engagement among older adults.

Because interactive interfaces and wearable technologies are inherently interconnected within digital health ecosystems, they were analyzed as components of a unified socio-technical system rather than as distinct categories. This integrative analytic stance enables the identification of cross-cutting mechanisms, such as how interface usability shapes the interpretation of wearable-generated feedback and how data visualization fosters motivation and adherence.

To enhance conceptual coherence, the thematic synthesis was guided by the Interface–Wearable Engagement Pathways (I-WEP) framework, which conceptualizes interactive interfaces and wearable technologies as a unified socio-technical system. Within this analytical lens, themes were grouped according to four interrelated pathways: usability and learnability, self-monitoring and adherence, motivational supports, and psychosocial empowerment, reflecting the mechanisms through which interface and wearable designs jointly shape health engagement among older adults. This framework thus provided not only a theoretical rationale for integrating the two domains but also an organizing structure for identifying cross-study regularities, tensions, and boundary conditions in the evidence.

## 3 Results

### 3.1 Study characteristics

A total of 48 studies were included in the final synthesis (Tables 4a–4e). To enhance interpretability and readability, the studies were categorized according to the type of intervention or sensor configuration, yielding five groups: (a) commercial wearable devices, (b) custom-built or experimental systems, (c) mobile health platforms and apps, (d) user-centered interface designs, and (e) immersive or alternative modalities. The 48 studies included in this review were conducted across diverse geographic regions, with Asia representing the largest proportion (21 studies, 43.8%), including nine studies from China (18–30), three from South Korea (31–33), and others from Japan (34), India (35, 36), Malaysia (37), and Saudi Arabia (38). North America contributed 17 studies (35.4%), predominantly from the United States (39–53) and two from Canada (54, 55). European countries accounted for ten studies (20.8%), with representation from Italy (56, 57), Germany (58), Finland (59), England (60), Ireland (61), Portugal (62), Slovenia (63), and multi-country collaborations involving Austria, Romania, and Portugal (64, 65). Notably, Africa was underrepresented with only one collaborative study involving Ethiopia and India (36), reflecting the concentration of wearable technology research in economically developed regions with advanced technological infrastructure and aging populations. The temporal distribution of the included studies reveals a concentration of recent publications, with the majority conducted between 2021 and 2025. Specifically, 2024 represented the most productive year with 15 studies published (31.3%) (19, 22, 27, 29, 32, 34, 36, 38–40, 43, 45, 53, 64, 65), followed by 2023 with 12 studies (25.0%) (21, 23, 25, 35, 37, 41, 49, 51, 54, 57, 59, 62). The year 2025 contributed 10 studies (20.8%) (18, 22–24, 26, 28, 30, 31, 42, 55), while 2022 yielded 6 publications (12.5%) (33, 44, 45, 50, 58, 61) and 2021 produced 5 studies (10.4%) (46, 47, 56, 60, 63). This distribution pattern demonstrates the rapidly growing research interest in wearable technology applications for older adult populations over the past 5 years, with approximately 77% of all studies being published in 2023–2024 alone, reflecting both the increasing availability of consumer-grade wearable devices and the heightened focus on digital health solutions for aging populations in the post-pandemic era.

**Table 4a:** Commercial wearable devices.

Author (Year)	Research design	Intervention type	Key findings
Ma et al., 2023 (19)	A four-week longitudinal mixed-method study with 20 older adults (aged 62–74, 75% female), mostly retired and smartphone users without prior wearable experience. Most participants had chronic conditions and lived with family.	Wearable devices (smartwatches or smart bracelets)	Participants’ awareness of health data increased over time, while notification/reminder features gained importance with continued use. Although behavioral changes such as reduced sedentary time were reported, overall activity levels remained stable. Adoption was influenced by utilitarian and hedonic values, as well as perceived wearability, ease of use, and credibility.
Shim et al., 2024 (39)	A cohort study combining data from the UK Biobank (*n* = 76,026; median age 58 years; 60% female) and NHANES (*n* = 3,371; median age 55 years; 55% female). Most participants were White, with common comorbidities including hypertension, cancer, and diabetes.	Wearable-based accelerometry data analysis	Wearable-derived cosinor age was identified as a robust biomarker of biological aging, associated with higher mortality and age-related disease risks, and declines in self-rated health and physical function. It outperformed activity-based metrics and underscored the role of circadian rhythm in aging processes.
Schmidle et al., 2022 (58)	An experimental cohort study involving 27 older adults (mean age 81.6 years, 67% female) from care institutions and the community. Participants were classified as robust, pre-frail, or frail based on physical performance and cognitive criteria (MMSE ≥ 24).	Smartwatch	Smartwatch-based kinematic analysis effectively distinguished frailty levels among older adults, with frail participants showing reduced agility, smoothness, and movement intensity. Differences were primarily linked to physiological rather than cognitive or sensorimotor factors.
Choma et al., 2023 (41)	An 18-month longitudinal study involving 113 community-dwelling adults aged ≥70 years (mean age 76.9; 77% female) using Fitbit Charge 2 devices. Participants had at least one fall risk factor and low physical activity levels but no neurocognitive impairment.	Physical activity monitors (Fitbit Charge 2)	Older adults experienced usability issues with activity monitors (e.g., wrist discomfort, device/app complexity, data interpretation). Frequent technical assistance was required, underscoring the need for user-centered design and structured support to sustain long-term use.
Ocagli et al., 2021 (56)	A multicenter prospective observational study involving 17 patients (median age 79; 59% female). Most had hypertension, with varying frailty levels and moderate functional limitation.	Smartwatch and smartphone for monitoring physical activity, rest heart rate, and sleep duration	Wearable devices were feasible and well accepted by older SAVR (Surgical Aortic Valve Replacement)and TAVR (Transcatheter Aortic Valve Replacement) patients (compliance ~75–80%), providing valuable behavioral data. Physical function declined after both procedures, more notably in TAVR patients, indicating a need for interventions to enhance post-procedure activity.
Kastelic et al., 2021 (63)	A controlled laboratory and free-living observational study involving healthy older adults (*n* = 28; mean age 74) without mobility or cognitive impairments. Participants wore consumer activity trackers and a research-grade monitor for performance comparison.	Wearing consumer-grade activity trackers and a research-grade activity monitor	Garmin trackers demonstrated high accuracy for step counts, and all tested devices accurately measured sleep time. Other movement behavior metrics showed low reliability and should be interpreted with caution.
Alpert et al., 2024 (43)	A qualitative study of 30 community-dwelling older adults (mean age 75; 53% female). Participants were generally healthy, digitally literate, and moderately active, with common conditions including hypertension and diabetes. Semi-structured interviews and usability surveys assessed feasibility and technology acceptance.	Apple Watch SE with the ROAMM app for real-time health assessment and mobility monitoring.	Most participants found the ROAMM smartwatch easy to use, with low perceived complexity and strong willingness for long-term use (73%). Usage was facilitated by interface clarity, health tracking appeal, and physician endorsement, while barriers included limited functionality, technical issues, and insufficient training. The system increased health awareness and showed potential to enhance patient engagement and clinical communication.
Zhang et al., 2025 (22)	A cross-sectional study using secondary data from HINTS 5 Cycle 4, including 1,521 U.S. adults aged ≥60 years (mean age 71). Common conditions included hypertension, diabetes, and heart disease.	Use of commercial wearable devices (e.g., Fitbit, Apple Watch, Garmin Vivofit) for health tracking	Wearable device use positively influenced older adults’ intention to share health data directly and indirectly through self-efficacy. Social support strengthened this relationship, with higher support linked to greater data-sharing intention.
Liang et al., 2024 (23)	A cross-sectional survey of 272 Chinese adults aged ≥65 years with at least 6 months of wearable health device (WHD) experience. Most preferred smart bracelets and had secondary or lower education; data were analyzed using PLS structural equation modeling.	Wearable Health Devices including smartwatches, smart bracelets, smart rings, smart glasses	Continuance intention toward wearable health devices was positively influenced by performance and effort expectancy, social factors, facilitating conditions, hedonic value, and perceived complexity, while technology anxiety and cost had negative effects. Habit and perceived risk were non-significant; the model explained 61% of variance.
Abouzahra et al., 2022 (44)	A qualitative Grounded Theory study involving 44 adults aged 65–75 from the U. S. and Canada with no prior wearable experience but smartphone ownership. Data were collected through pre/post-use interviews and device usage analysis.	Fitbit Charge 2 wearable device	Older adults used wearable devices for activity tracking, planning, and self-improvement. Perceived physical capability, technological readiness, and social influence facilitated adoption and continued use, while information quality and adaptability were key to effective engagement through user–device interaction.
Arnaert et al., 2023 (54)	A qualitative descriptive study guided by the UTAUT2 model involving 10 community-dwelling older adults with COPD (mean age 71.6). Participants, mostly with severe symptoms (GOLD level D), were interviewed to explore technology acceptance in telehealth contexts.	Smartwatch (Apple Watch Series 6), digital fingertip pulse oximete, Telemonitoring platform	Older adults with COPD (chronic obstructive pulmonary disease) perceived the Apple Watch mainly as a medical tool, noting potential benefits for well-being and physical activity. Social influence and family support strongly facilitated use, while limited tech confidence and device ownership constrained engagement. Reliability issues with SpO₂ readings reduced trust compared with traditional oximeters.
Batsis et al., 2021 (60)	A 12-week single-arm pilot feasibility study with 28 participants (mean age 72.9).	Wearable Device: Fitbit	Wearable-based weight loss intervention showed high feasibility and acceptability among obese older adults in rural areas, leading to significant weight loss and improved physical function. Participants viewed Fitbit use positively, though community-level implementation remained challenging.
Chung et al., 2023 (49)	A mixed-methods study with 30 community-dwelling older adults (mean age 67.4; 63% female) who used GPS-enabled smartwatches for 3 days. Usability surveys and interviews explored user perceptions, barriers, and facilitators to smartwatch use.	Fitbit Surge smartwatch	Most older adults, particularly those with chronic conditions, showed positive attitudes and willingness to use wearable devices for health monitoring. Device design, comfort, and usability strongly influenced acceptance, while privacy concerns were minimal. Participants valued broader health tracking and clearer guidance to enhance engagement and accuracy.
Nascimento et al., 2025 (55)	This study explored the best wearable device and placement for assessing mobility in hospitalized older adults. In Experiment 1 (*N* = 25, mean age 79.6 years, 65% female), four devices were compared at different body positions during mobility tasks. In Experiment 2 (*N* = 30, mean age 81.4 years, 80% female), the ActiGraph was tested on the wrist, thigh, and ankle during activity tests and 24-h monitoring to evaluate its feasibility and comfort.	A variety of wearable devices	The study found that low activity levels among hospitalized older adults were often due to care models rather than illness, leading to functional decline. Wearable devices, especially the ActiGraph wGT3X-BT, effectively monitored activity and mobility patterns, with thigh-worn sensors accurately identifying sedentary behavior and ankle-worn sensors improving step detection and posture recognition. Overall, the ActiGraph showed the best performance in data integrity, battery life, and usability. Most patients were willing to wear the devices, preferring ankle or waist placement. Combining thigh- and ankle-worn devices was recommended to enhance monitoring accuracy and support personalized mobility care.
Wu et al., 2025 (26)	This quasi-experimental study involved 80 healthy older adults (40 males, 40 females, aged 65+), randomly assigned to an intervention or control group. The intervention group participated in a 12-week structured walking program (30 min per day, 5 days per week, at 100 steps per minute) assisted by wearable devices. Step count and calorie data were automatically synchronized, and body composition was assessed using bioelectrical impedance analysis. Muscle strength, walking speed, grip strength, and sarcopenia risk were evaluated, with statistical analyses conducted to determine the program’s effects and related associations.	Wearable devices: Garmin Vivosmart HR and Apple Watch	The 12-week walking intervention significantly increased physical activity among older adults, with average daily steps reaching 6,119 for males and 5,709 for females on workdays. Participants in the intervention group showed marked improvements in body composition, including increased skeletal muscle mass, lean body mass, basal metabolic rate, and appendicular skeletal muscle index, alongside reductions in body fat and waist-to-hip ratio. Muscle strength and mobility also improved significantly, indicating that the program effectively enhanced muscle health and reduced the risk of sarcopenia.
Tanaka et al., 2024 (34)	This study involved 7 older adults (mean age 75.0 ± 7.3 years) and 14 healthcare providers (mean experience 21.1 ± 10.1 years) to explore perceptions of wearable health technologies in Japan. Researchers conducted focus groups and interviews—both online and in person—to discuss health concepts and wearable device use. Using grounded theory and iterative coding, the analysis was collaboratively refined until data saturation, ensuring rigor through multi-researcher verification and regular discussions.	wearable technology	This study explored factors influencing older adults’ health and the potential role of wearable technology. It found that social connection and personal motivation were key to maintaining health, while social isolation increased risks. Healthcare providers emphasized that sustaining life goals and exercise motivation was more crucial than nutrition or activity alone. Wearable technologies were viewed as promising for early risk detection, chronic disease management, and continuous home monitoring, but challenges remained—such as usability difficulties for older adults, limited relevance for healthy individuals, and concerns about cost, reliability, data privacy, and reduced family contact.
Silveira et al., 2021 (51)	This cross-sectional study included 440 older adults with multiple sclerosis (MS) recruited through the National MS Society, of whom 28% used fitness trackers. Participants aged 60 and above completed an online survey collecting demographic data, disability level (PDDS), tracker use, and physical activity measured by the Godin Leisure-Time Exercise Questionnaire (GLTEQ), to examine the relationship between tracker use and activity levels.	Fitness tracker	This study found that fitness tracker use among older adults with multiple sclerosis was significantly associated with higher levels of physical activity. Tracker users reported greater total and health-promoting activity, and step counts correlated strongly with GLTEQ scores. As physical activity benefits mobility and overall health in MS, fitness trackers may help enhance independence and well-being, though further research is needed to confirm effects in less active patients.
Martinato et al., 2021 (57)	This non-interventional field study involved 49 older adults (median age = 75 years, range = 70–90) from northeastern Italy to evaluate the accuracy of the Garmin vívoactive HR smartwatch in measuring step counts. Participants, all able to walk independently, performed a 150-meter walk at their natural pace in real-life environments such as local markets to assess the device’s validity for tracking everyday physical activity in older adults.	Commercially available smartwatch	This study demonstrated that wrist-worn wearable devices, such as the Garmin vívoactive HR, accurately measured older adults’ daily physical activity in real-life settings, showing excellent agreement with manual counts (ICC = 0.98). The findings suggest these devices can reliably monitor activity levels and support healthy behaviors in postoperative rehabilitation and chronic disease management, offering potential for clinical use and integration into health care monitoring systems.
Low et al., 2024 (52)	This observational study recruited 40 elderly cancer survivors (mean age 73 years; 50% female; 70% recently treated) to examine the relationship between physical function and digital health data. Participants completed standardized questionnaires and physical performance tests while continuously wearing Fitbit Inspire 3 devices for 4 weeks, with additional passive location data collected via the AWARE smartphone app.	Wearable devices (Fitbit Inspire 3) and smartphone sensor data	This study found that wearable device data—such as activity volume, fragmentation, and peak gait cadence—were strongly correlated with clinical measures of physical function in elderly cancer survivors, even after adjusting for age and comorbidities. Smartphone mobility data showed weaker associations, suggesting that wearable devices are more reliable supplementary tools for functional assessment.
Wong et al., 2024 (29)	This qualitative study involved 22 community-dwelling older adults (aged 62–78 years; 77% female) to explore their experiences using wearable monitoring devices. Participants received a health monitoring kit and training, followed by focus group discussions analyzed through inductive comparative methods to identify key themes on usability and user experience.	Wearable Monitoring Devices	This study found that wearable monitoring devices helped older adults conveniently track health indicators, share data with healthcare providers, and stay motivated for physical activity, enhancing their engagement in self-health management. However, users faced challenges such as learning difficulties, bulky design, short battery life, and occasional data inaccuracies.
Jiang et al., 202 (63)	This secondary analysis used data from 1,484 U. S. adults aged 65–98 years (mean age 73.8) from the HINTS 5-Cycle 3 survey, of whom 74% had cardiovascular disease or related risk factors.	Commercial wearable electronic devices	This study found that 16% of older adults used wearable devices, and these users were more likely to meet physical activity guidelines for moderate and enhanced exercise. Device use was associated with higher income, better self-rated health, and greater enjoyment of exercise, with particularly strong benefits among those with cardiovascular disease or related risks.
Lee et al., 2021 (30)	This 12-month prospective cohort study included 319 community-dwelling adults from Taipei (mean age = 64.9 ± 6.6 years, range = 50–85) who were free of end-stage disease or active cancer and able to provide informed consent.	Wearable device (“Lifebeat,” NeuroSky, Inc., CA, USA)	The study found that active wearable device users had faster walking speeds, higher IGF-1 levels, and better cognitive performance than inactive users. Each additional 1,000 daily steps was linked to a 0.03 m/s increase in walking speed, and an average of 7,008 steps per day was identified as the optimal threshold to prevent mobility decline.
Neves et al., 2023 (62)	This open-label randomized controlled trial planned to recruit 100 adults aged over 65 with multimorbidity (≥2 chronic conditions) from outpatient clinics in Lisbon, Portugal, all of whom owned a smartphone.	Fitbit Sense smartwatch and Withings sleep analysis mattress	This study outlines a randomized controlled trial comparing a wearable sleep mat intervention with a control group in older adults with multimorbidity. The primary outcome is the 6-month change in physical activity (IPAQ-SF), with secondary measures including frailty, body composition, quality of life, biopsychosocial status, sleep quality, heart rate, step count, and unplanned hospitalizations.

**Table 4b:** Custom-built/experimental systems.

Author (Year)	Research design	Intervention type	Key findings
Wang et al., 2025 (18)	A controlled experimental study involving 10 participants conducted a 24-h synchronous multi-user wear test. Participants were allowed free movement during testing, with recorded variations in heart rate, respiration, and body temperature, as well as occasional sinus bradycardia and premature ventricular contractions.	Wearable waistband system	The wearable waistband system enabled continuous, real-time monitoring of respiration, ECG, and body temperature across multiple users. It effectively detected physiological variations and abnormalities (e.g., sinus bradycardia, premature ventricular contractions), demonstrating stability during activity and potential for intelligent healthcare and elderly care applications.
Joymangul et al., 2024 (64)	A pilot study involving 112 older adults (mean age 72.7 years, 66% female) from three European regions (Romania, Portugal, Italy). Most participants were retired, married, and living in rural areas, with no major physical or cognitive impairments.	IoT-based wearable and mHealth devices	The AGAPE platform increased physical activity and social engagement among older adults through wearable-assisted interventions. Usability and technostress levels varied across user profiles, highlighting the need for personalized, user-centered design to support active and healthy aging.
Ghosh et al., 2023 (35)	Experimental study involving elderly residents of assisted living facilities, focusing on federated learning–based IoMT healthcare framework development. Participants had varied chronic conditions (e.g., diabetes, asthma, hypertension), with demographic and environmental factors also considered.	FEEL framework (federated learning IoMT)	The FEEL framework achieved high accuracy in activity monitoring, fall detection, and health recommendation tasks, outperforming baseline models by 10–16%. It effectively addressed data sparsity and user diversity, offering privacy-preserving and personalized IoMT healthcare solutions for older adults.
Mathew et al., 2024 (40)	A randomized controlled trial involving 104 adults aged 50–87 years with prior ischemic stroke or transient ischemic attack. Most participants were White and had comorbid cardiovascular conditions such as hypertension, diabetes, and hyperlipidemia.	A custom-built smartwatch-app dyad, the Pulsewatch system	Smartwatch use for atrial fibrillation monitoring was well accepted among stroke survivors, with no major age-related differences in anxiety, engagement, or physical health perception. A slight decline in perceived mental health was observed in the 61–69 age group compared to younger participants.
Hsu et al., 2023 (20)	An evaluation study using DEMATEL and ANP methods with nine multidisciplinary experts (architecture, urban planning, information engineering, and healthcare). Empirical analysis covered 30 elderly care institutions across 10 districts in Suzhou, China.	Smart monitoring system for nursing homes	The study identified key performance indicators for smart monitoring systems in elderly care, including emergency alerting, system efficiency, data accuracy, and safety protection. Sensor technology was highlighted as essential for effective motion and fall detection, vital sign monitoring, and emergency response.
Um et al., 2025 (31)	Experimental validation study using overnight recordings from a single volunteer to train and test AI models against standard polysomnography (PSG).	Wearable device integrated with stretchable transparent electrodes	The AI-based wearable device achieved 73% accuracy (F1 = 0.72) in sleep stage classification, comparable to standard PSG. With stretchable transparent electrodes, it enables compact, multisignal home sleep monitoring and supports personalized sleep care.
Veiciu et al., 2023 (65)	A qualitative user-centered design study involving 30 older adults (aged 65–80+) and 32 informal caregivers from Austria, Italy, and Romania. Participants were mostly retired or employed caregivers, recruited through local organizations via structured interviews and questionnaires.	CAREUP platform (wearable + environmental sensors + gamified care app)	Most older adults valued self-monitoring and were willing to use wearable sensors, especially for real-time alerts and personalized care plans. Barriers included limited technology experience, usability challenges, and data reliability concerns. Both older adults and caregivers recognized benefits in supporting care and reducing stress, with a preference for professional guidance during use.
Paramasivam et al., 2024 (36)	An experimental study with 120 healthy adults aged 60–70 performing simulated daily activities for accelerometer-based data collection. The study evaluated a wearable prototype and deep learning model for ADL recognition and edge computing performance.	AI-Enabled Wearable Fall Detection Device, Deep Learning Models, Edge Computing Devices, User Interface & Notification System	The CNN-LSTM with attention model achieved the highest accuracy (97%) for ADL recognition. The wearable device effectively detected five activities and enabled real-time caregiver alerts. The Jetson Nano provided fastest processing, while Raspberry Pi 4 offered best cost-performance. FIR filtering improved signal quality, though activity coverage remained limited.
Mardini et al., 2021 (46)	A demonstration study with 19 older adults (mean age 73.1) diagnosed with knee osteoarthritis. Participants wore a smartwatch for ~13 days using the ROAMM platform to collect EMA pain and GPS data, analyzed with multilevel modeling to assess pain-related effects on life-space mobility.	Custom-designed smartwatch platform named Real-time Online Assessment and Mobility Monitor (ROAMM)	Higher daily pain intensity was associated with reduced life-space mobility within individuals, including smaller excursion size and distance traveled, while between-person effects were non-significant. The ROAMM smartwatch platform reliably captured EMA pain and GPS mobility data with an 82% response rate.
Lin et al., 2023 (25)	A developmental study involving 12 participants (7 young, 5 middle-aged) to design and validate a chest-worn wearable and IoT-based fall detection system. The study compared wearable, vision-based, and environmental sensing methods to evaluate their relative strengths and limitations.	A fall recognition and verification system based on a chest-worn wearable device.	A chest-worn wearable and IoT-based fall detection system demonstrated excellent performance in simulated falls, achieving sensitivity, specificity, and accuracy above 0.94. The system provided real-time posture monitoring, instant alerts, and long-term data analysis to support personalized fall prevention. Limitations included potential skin allergies from adhesives and limited 24-h battery life requiring daily charging or dual-device use.
Lin et al., 2024 (27)	This study implemented a Smart Technology-Assisted (STA) home care program for 12 elderly participants with dementia or recovering from hip fractures and their caregivers. The program featured a washable smart vest with embedded sensors and an HMM algorithm to monitor activity, gait, posture, and falls, with data transmitted to a mobile app and cloud platform. Additional home sensors, including exit alarms, smoke detectors, and emergency buttons, enhanced safety. Participants wore the vest at least 4 days a week for 3 months, supported by regular nurse visits and phone follow-ups. Feedback was analyzed thematically to evaluate usability and effectiveness.	Smart vest with sensors and HMM algorithm	The Smart Technology-Assisted (STA) home care program aimed to support elderly patients with dementia or hip fracture recovery and their caregivers by using a sensor-embedded smart vest connected to a mobile app and cloud platform for remote monitoring and fall detection. The program enhanced care quality through personalized alerts, home environment assessments, and regular nurse follow-ups, helping reduce caregiver burden and potentially improving patient outcomes. Although technical issues such as unstable networks and sensor malfunctions occurred, they were effectively resolved through system adjustments and improved power management.
Wang et al., 2025 (28)	This randomized controlled trial recruited 400 older adults with essential hypertension, randomly assigning 200 each to an experimental and a control group. From September 2022 to November 2023, the experimental group used a wearable-based chronic disease management platform with Bluetooth-enabled blood pressure monitoring, cloud data upload, and real-time personalized interventions, while the control group received standard community health management.	A chronic disease management platform based on wearable devices and traditional methods	This study found that the wearable-based chronic disease management platform significantly improved health outcomes in elderly patients with hypertension. Compared with traditional management, the experimental group achieved higher blood pressure control (80.5% vs. 60.5%), better medication adherence, improved quality of life across physical, social, and emotional domains, and notable gains in hypertension knowledge and self-management efficacy.

**Table 4c:** Mobile health platforms and apps.

Author (Year)	Research design	Intervention type	Key findings
Alnanih et al., 2023 (38)	A mixed-methods study involving 602 older Saudis (≥60 years; 63% female) using surveys, interviews, and usability testing. Most participants were retired or unemployed, with varied education and income levels; medication non-adherence was common (79%).	Design and evaluation of a mobile application prototype for medication adherence tailored to older Saudis.	The mobile application improved medication adherence among older Saudis, showing high effectiveness and error safety but lower productivity due to age-related factors. Both independent and dependent users reported high satisfaction, emphasizing the importance of designing interfaces aligned with older adults’ cognitive and physical characteristics.
Kari et al., 2023 (59)	A follow-up study involving 241 adults aged ≥60 years (mean age 69.8; 62% female). Most participants were married and vocationally educated, with 64–80% meeting WHO physical activity recommendations at baseline.	Use of a mobile physical activity tracker application	Use of a physical activity (PA) tracker app significantly increased walking, moderate, and total PA levels among older adults, with effects sustained over 24 months. No significant change was observed in sedentary behavior.
Villalobos et al., 2022 (45)	An observational mixed-methods study involving 16 older adults with advanced heart failure (mean age 76) and 10 caregivers (mean age 71). Participants completed qualitative interviews and quantitative usability assessments (uMARS) to evaluate a mobile health application for home-based heart failure care.	Hardware: Tablet-based tools, smartwatch Software: Web-based system portal, mobile app.	The Convoy-Pal app showed good usability and acceptability (uMARS = 3.96/5), with information quality rated highest and engagement lowest. Participants valued data sharing, goal setting, and monitoring tools, suggesting potential to enhance palliative care access and self-management. Feedback highlighted needs for added features, simplified visuals, and reduced caregiver burden.
Song et al., 2024 (32)	A single-arm, non-randomized living lab pilot study with 25 older adults (mean age 76.4; 76% female).	A digital mental health monitoring platform	A 6-week digital platform and community care intervention among 25 older adults significantly improved depressive symptoms and sleep quality. Daily sleep efficiency and fragmentation predicted next-day depressive symptoms, but heart rate variability and activity did not. Despite high satisfaction with the chatbot, smartwatch usability issues persisted throughout the study.
Seok et al., 2022 (33)	A 5-month pre-experimental mixed-methods study was conducted among 111 older adults living alone (mean age = 77.3 ± 3.8 years; 78.6% female). Participants used the TouchCare system—a combination of touch tag sensors, a wearable watch, a mobile application, and an AI assistant (“Suni”). Researchers installed the system and provided training before use. After the intervention, 91 participants completed a satisfaction survey, and 22 provided pre- and post-data on demographic, medical, behavioral, psychological, and physiological indicators.	A technologically-based health monitoring and management intervention based on the TouchCare system.	The TouchCare system significantly improved older adults’ nutritional status and fall efficacy, with positive but non-significant trends in frailty, depression, pain, cognition, and physical function. Use of the system was also associated with increased physical activity, improved gait, healthier eating patterns, and more frequent outings. Over half of participants were satisfied with TouchCare, citing benefits such as reduced loneliness, greater emotional stability, and enhanced care quality. The AI assistant “Suni” was viewed as supportive for daily engagement. However, some participants reported limited practicality and discomfort with the wearable watch. Overall, the system showed potential as a cost-effective virtual care tool for older adults living alone, though larger studies are needed to confirm its long-term health and behavioral benefits.

**Table 4d:** User-centered interface designs.

Author (Year)	Research design	Intervention type	Key findings
Liu et al., 2025 (21)	A mixed-methods study of 108 older adults (≥60 years) from three Beijing nursing homes, combining interviews, questionnaires, and visual simulation experiments. Participants were long-term smartphone users without color vision disorders; the study applied the HSL model to examine app interface color design preferences.	Analyzing interfaces from 10 elderly-oriented Chinese mobile apps.	Older adults preferred app interfaces with warm hues, medium saturation, and high lightness, which enhanced visual comfort and emotional well-being. Cool or low-lightness tones induced negative emotions, while abstract icons and poor layout reduced usability. Warm colors (e.g., orange, yellow) and clear graphical elements improved visual comfort and operational smoothness.
Allah et al., 2023 (37)	A comparative usability study with 20 adults aged 60–74 from Malaysia, Libya, Yemen, and Egypt, comparing an adaptable web search interface with Google’s default UI. Participants were mostly male, educated, and experienced internet users, with common conditions including visual impairment and diabetes.	Adaptable Web Search UI Prototype for the Elderly	The adaptable web search UI prototype outperformed Google’s default interface, showing higher usability (SUS = 90.3 vs. 65.3), faster task completion, fewer errors, and better learnability. Older adults adapted quickly despite unfamiliarity, indicating strong usability and accessibility advantages.
Ma et al., 2024 (24)	A laboratory experiment with 23 older adults (mean age 66.7; 65% female) using a 2 × 2 within-subject design comparing information amount (simple vs. rich) and interface type (UnitDesign vs. Reference). All participants were smartphone users, and over half had prior wearable device experience.	UnitDesign Interface (wearable information display design approach) and Reference Interface (mainstream commercial smartwatch design)	UnitDesign improved usability under rich data conditions, reducing search time and increasing satisfaction and perceived clarity, though comprehension accuracy remained unchanged. Most participants preferred UnitDesign and richer information for deeper understanding, while some favored simpler displays to minimize cognitive load.
Wu et al., 2021 (47)	A within-subjects mixed-methods study with 30 older adults (mean age 71) evaluating two telepresence UIs—Presence (control) and InTouch (experimental)—in a simulated virtual environment. Quantitative (SUS, TAM, performance data) and qualitative (interviews) measures assessed usability and user experience.	Telepresence user interfaces, Unity-based simulated virtual environment	The InTouch interface outperformed Presence in usability (higher SUS and ease-of-use scores), navigation accuracy, and perceived privacy. Most participants (97%) preferred InTouch, citing greater usefulness and smoother control. Telepresence robots were widely accepted, viewed as beneficial for social connection and independent living support.
Cotter et al., 2024 (48)	An online randomized experimental study with 1,378 participants (50.6% female), primarily aged 40–74 and predominantly White or Black.	Different designs of data visualization dashboards for influenza vaccination	All dashboard designs increased older adults’ perceived susceptibility to influenza, though vaccination intention remained unchanged. Interactive dashboards impaired information recall, while explanatory text significantly improved memory, especially among older participants.

**Table 4e:** Immersive and alternative modalities.

Author (Year)	Research design	Intervention type	Key findings
Lee et al., 2025 (42)	A pilot observational cohort study involving 30 community-dwelling adults aged 65–80 years (mean age 71.8), including both men and women. Participants were generally healthy, though five had reported falls in the previous 6 months.	Use of wrist-worn voice recorders	Wrist-worn voice recorders effectively captured real-time circumstances of balance loss among older adults, offering richer contextual data than memory recall. Most incidents occurred at home or in the afternoon, typically while walking or tripping, without resulting in falls.
Flynn et al., 2022 (61)	This study used a Participatory Action Research (PAR) approach to explore the applicability of virtual reality (VR) for community-dwelling older adults with dementia and their caregivers. Nine patient-caregiver pairs participated, with patients aged 59–80+. Using the VR FOUNDATIONS technology probe, participants engaged in 20–25 min home-based VR sessions featuring interactive environments adapted to their abilities. Researchers observed behaviors, conducted post-session interviews, and analyzed feedback through reflective thematic analysis with NVivo, integrating insights from both patients and caregivers to inform future participatory VR design.	A virtual reality technology probe named VR FOUNDATIONS	The study found that the VR FOUNDATIONS system had multiple positive effects on dementia care. Its multi-sensory stimulation enhanced patients’ alertness, engagement, and emotional well-being, with many showing greater focus, relaxation, and social interaction, though a few found the experience overstimulating. The system’s passive and interactive environments allowed users to choose their level of engagement, and its hardware and controls were generally easy to use. Verbal guidance, gradual transitions, and caregiver presence boosted user confidence, while direct experience reduced fear of new technology and increased interest in future VR use.
Orlofsky et al., 2022 (50)	This qualitative descriptive study explored factors influencing the adoption and use of the Amazon Alexa voice assistant among 12 home-dwelling older adults aged 67–92 years (mean age = 76.83; 8 females, 4 males). Participants, all with at least 6 months of experience using Alexa, took part in recorded and transcribed interviews that were participant-verified for accuracy. Using inductive thematic analysis and manual coding, key patterns were identified, and the rigor of analysis was ensured through external expert review and reflective journaling.	Voice-activated personal assistants (VAPAs): Amazon’s Alexa	The study found that while older adults did not view Alexa as essential, its use provided social support and daily life assistance, potentially benefiting aging in place. Most participants received the device as a gift and relied on others for setup, with limited training leading to underuse of functions. They mainly used Alexa for entertainment, reminders, and companionship, rarely for emergency contact. Despite privacy and voice recognition concerns, users were generally satisfied and felt Alexa improved their quality of life and reduced loneliness.

The included studies employed diverse methodological approaches to investigate wearable technology in older adult populations. Qualitative designs were utilized in 7 studies (14.6%) (29, 34, 37, 43, 44, 54, 61), exploring user experiences, acceptance, and perspectives through interviews and focus groups. Observational studies, including cross-sectional surveys and cohort designs, comprised 12 studies (25%) (30, 39, 40, 42, 45, 46, 51, 53, 55–57, 63), focusing on feasibility, validity, and correlation analyses. Experimental research designs were employed in 9 studies (18.8%), which varied in their degree of control and random assignment. Among these, 4 studies (8.3%) (28, 40, 48, 62) implemented randomized controlled trials (RCTs) with full randomization and control groups, while 5 studies (10.4%) (24, 26, 35, 36, 58) utilized quasi-experimental or experimental designs (non RCTs) to examine intervention effects. Feasibility and pilot studies represented 8 studies (16.7%) (18, 31–33, 52, 55, 58, 60), testing the practicality and preliminary efficacy of interventions. Mixed-methods designs, combining qualitative and quantitative approaches, were employed in 11 studies (22.9%) (19, 21, 25, 27, 38, 41, 45, 47, 49, 64, 65), providing comprehensive insights into both user experiences and measurable outcomes. Additionally, 3 studies (6.3%) (18, 20, 35) focused on system development and validation without traditional clinical trial designs.

The studies included diverse older adult populations with varying demographic and clinical profiles. Sample sizes ranged from small feasibility studies with fewer than 30 participants (19, 42, 43, 45, 49, 58) to large-scale population-based studies exceeding 1,000 participants (22, 39, 48, 53). The mean age of participants typically ranged from 65 to 80 years, with some studies including middle-aged adults from 50 years onward (30, 40). Gender distribution varied across studies, with several reporting predominantly female samples (29, 32, 41, 51), while others maintained balanced gender representation (28, 44). Many studies focused on community-dwelling older adults (19, 29, 30, 41, 44, 46, 49, 59), while others targeted specific clinical populations including those with chronic conditions such as hypertension (28), COPD (54), multiple sclerosis (51), cancer survivors (52), heart failure (45), dementia (27, 61), and frailty (55, 56, 58). Digital literacy levels varied considerably, with some studies explicitly requiring smartphone ownership and basic technological competence (28, 29, 32, 62), while others reported varying levels of familiarity with technology among participants (19, 34, 41, 44, 54).

Caregiver involvement was explicitly documented in 13 studies (27.1%) (25, 27, 29, 32–34, 38, 45, 54, 56, 61, 64, 65), highlighting their critical roles as intermediaries between older adults and healthcare systems. Caregivers’ responsibilities varied substantially across studies, encompassing technical support functions such as device setup, synchronization, charging, and troubleshooting (27, 54, 56), health monitoring activities including reviewing health data, responding to alerts, and tracking vital signs (25, 29, 32, 33), and active participation in decision-making processes regarding care interventions (27, 45, 64, 65). In five studies, caregivers were predominantly family members, including spouses, adult children, or other relatives (34, 45, 54, 61, 65), while others involved formal caregivers such as community health workers, nurses, and social workers (29, 32). The intensity of caregiver involvement ranged from minimal remote monitoring (25, 33) to essential hands-on support, with one study reporting that only one out of 17 elderly patients could independently manage device tasks, while all others required caregiver assistance for synchronization and charging (56). Qualitative findings revealed that caregivers themselves experienced both benefits and burdens from technology use; they appreciated the reassurance provided by continuous monitoring (29, 32, 33) but also expressed concerns about data reliability, emergency response capabilities, and the emotional toll of caregiving responsibilities (27, 34, 65). Some studies identified caregivers as dual beneficiaries of wearable technology, with 5 out of 7 elderly participants in one Japanese study also serving as caregivers for their own spouses or parents, highlighting their interest in monitoring their own health alongside that of care recipients (34). However, the majority of studies (35 studies, 72.9%) did not explicitly mention or include caregivers in their interventions (18–24, 26, 28, 30, 31, 35–37, 39–44, 46–53, 55, 57–60, 62, 63), suggesting a significant gap in acknowledging the interconnected nature of older adult care and the potential role of caregivers as facilitators of technology adoption and sustained use.

Interventions and technologies covered a broad spectrum of hardware and software configurations. Commonly evaluated technologies were consumer wrist-worn devices (smartwatches, fitness bands) used alone or paired with mobile apps, such as step or activity trackers and atrial fibrillation (AF)-detection smartwatch-app dyads (40, 59), bespoke wearable systems and sensor arrays, including waistband systems, smart socks, inertial measurement unit (IMU)-based fall detectors (18, 36, 64), integrated Internet of Things (IoT) or mobile health (mHealth) platforms combining wearables with cloud dashboards and caregiver portals (37, 64), and interactive user interfaces or information-display prototypes designed specifically for older users, such as UnitDesign information displays, color or contrast studies (24). A small subset of studies also explored immersive or alternative interfaces such as simplified Virtual Reality (VR) probes for people with dementia (40). This typological grouping (Tables 4a–4e) clarifies the technological diversity across studies and facilitates comparison of sensor functions, interface mechanisms, and user-centered outcomes.

The studies reported diverse outcomes demonstrating both the potential and challenges of wearable technology interventions for older adults. Physical activity outcomes were frequently assessed, with several studies reporting significant improvements in step counts, walking time, and moderate-to-vigorous physical activity levels (26, 30, 53, 59, 64). Specifically, wearable-assisted interventions led to increased daily step counts from baseline to follow-up (26, 59), with one study identifying 7,008 steps as an optimal threshold for preventing walking speed decline (30). Health monitoring capabilities were validated across multiple domains, including fall detection accuracy (25, 35, 36), cardiovascular monitoring (28, 40), sleep quality assessment (31, 32, 62), and mobility tracking (46, 52, 55). Clinical outcomes demonstrated improvements in disease management, with one RCT reporting higher blood pressure control rates in the intervention group (80.5% vs. 60.5%) (28), while another showed significant improvements in depressive symptoms (*p* = 0.048) and sleep quality (*p* = 0.02) among socially vulnerable older adults (32). Body composition improvements were documented in a wearable-assisted walking program, with skeletal muscle mass increasing by 5.5% in men and 3.7% in women (26). Usability and acceptance varied considerably, with several studies identifying barriers including device complexity (29, 41, 49), inadequate training (32, 43, 50), technical difficulties (27, 41, 55), privacy concerns (23, 34, 49), and cost limitations (23, 51, 53). Facilitators included caregiver support (27, 32, 54, 56, 61), social influence (22, 44, 64), user-friendly design (24, 33, 38, 43), and perceived health benefits (19, 29, 44).

A detailed yet categorized overview of all studies—including author, year, research design, intervention type, and key findings—is provided in Tables 4a–4e. These characteristics contextualize the subsequent synthesis, which is organized along the four I-WEP pathways to explain how interface–wearable systems shape engagement outcomes.

### 3.2 Types of interactive interfaces and wearable technologies (RQ1)

Thematic analysis of the extracted texts yielded five overarching themes that capture the types of interactive interfaces and wearable technologies investigated in the included studies. These themes encompass both widely adopted consumer devices and more experimental systems tailored for older adults. The themes are summarized in Table 5 and elaborated below with supporting evidence from the reviewed literature. Consistent with the I-WEP analytical lens, each theme is mapped to one or more pathways (P1 usability/learnability; P2 self-monitoring/adherence; P3 motivational supports; P4 psychosocial empowerment).

**Table 5:** Themes identified from the thematic analysis of RQ1 (types of interactive interfaces and wearable technologies).

Theme	Features/Codes	Representative studies (ref. no.)
Commercial wearable devices	Smartwatches, fitness trackers; monitoring of steps, sleep; paired with mobile apps	(19, 22, 23, 26, 29, 30, 34, 39, 41, 43, 44, 49, 51–58, 60, 62, 63)
Custom-built wearable systems	Smart socks, waistbands, IMU-based sensors; fall detection, gait/rehabilitation monitoring	(18, 20, 25, 27, 28, 31, 35, 36, 40, 46, 64, 65)
Mobile apps and mHealth platforms	Standalone or companion apps; dashboards, reminders, caregiver links, self-management tools	(19, 21, 24, 28, 29, 32, 33, 37, 38, 45, 48, 55, 59, 62, 64)
Interactive interface designs	Simplified navigation, larger buttons, high-contrast displays, older-adult–friendly HCI (Human-Computer Interaction) adaptations	(21, 24, 37, 47, 48)
Immersive/alternative modalities	Voice-assisted interaction, VR environments for cognitive/social support	(42, 50, 61)

#### 3.2.1 Theme 1: commercial wearable devices

Commercial wearable devices, most prominently smartwatches and fitness trackers, supported continuous monitoring of physical activity, cardiovascular parameters, and sleep across 23 studies (19, 22, 23, 26, 29, 30, 34, 39, 41, 43, 44, 49, 51–58, 60, 62, 63). Specific devices included Fitbit models (41, 44, 49, 51–53, 55, 60), Apple Watch (22, 26, 43, 51, 53, 54), and Garmin devices (26, 30, 51, 53, 56, 57, 63). In 8 studies (23, 28, 29, 32, 41, 44, 59, 62), these wearables were paired with mobile applications to enhance feedback and self-management capabilities. Within the I-WEP framework, this theme corresponds primarily to Pathway 2, emphasizing self-monitoring and adherence through continuous tracking, and secondarily to Pathway 3 when motivational features such as goal-setting or reminders were integrated.

#### 3.2.2 Theme 2: custom-built wearable systems

Custom-built wearable systems, including sensor-embedded textiles, waist-mounted belts, chest-worn devices, and inertial measurement units, were designed for fall detection or gait analysis in 12 studies (18, 20, 25, 27, 28, 31, 35, 36, 40, 46, 64, 65). These prototypes emphasized medical-grade precision and multi-sensor integration, aiming to address specific clinical needs in older populations such as rehabilitation monitoring (27, 64). However, 4 studies (25, 27, 36, 64) noted challenges in scalability and everyday adoption compared to consumer-grade devices. This theme aligns with Pathway 2 by delivering high-fidelity feedback for condition-specific self-management, while also illustrating a recurring challenge within Pathway 1, as enhanced precision often increases the burden of setup, maintenance, and comfort (18, 27).

#### 3.2.3 Theme 3: mobile health applications and integrated mHealth platforms

Mobile applications and mHealth platforms functioned either as independent health-management tools or as companion applications to wearable devices in 15 studies (19, 21, 24, 28, 29, 32, 33, 37, 38, 45, 48, 55, 59, 62, 64). These systems frequently offered interactive dashboards (37, 48), reminders (33, 38, 59), progress tracking (19, 44, 59), and caregiver communication features (29, 32, 33, 45, 64), thereby promoting older adults’ active participation in self-management. Integration between hardware and software was identified as critical for sustaining engagement in 6 studies (28, 32, 45, 59, 62, 64). According to the I-WEP framework, these systems primarily address Pathway 2 by supporting actionable feedback loops and Pathway 3 by providing motivational reinforcement. When caregiver portals or social communication functions were incorporated (32, 33, 45, 64), they also contributed to Pathway 4 by enhancing reassurance and perceived support.

#### 3.2.4 Theme 4: interface designs optimized for older adults

Studies focusing on interface design emphasized human–computer interaction adaptations such as larger buttons, simplified navigation, and high-contrast displays in 5 studies (21, 24, 37, 47, 48). These design adjustments addressed sensory and cognitive barriers to technology adoption (21, 24) and underscored the pivotal role of usability in shaping health engagement outcomes (37, 47). Specifically, 3 studies (6.3%) (21, 24, 37) reported improved user satisfaction and task completion efficiency through age-tailored visual design. This theme represents the central mechanism of Pathway 1, which focuses on usability and learnability. Improvements in perceptual clarity and cognitive simplicity can subsequently facilitate Pathway 2 processes by reducing the mental effort required for self-monitoring and daily use (24).

#### 3.2.5 Theme 5: immersive and alternative interaction modalities

Emerging modalities, including voice-assisted systems (50), haptic feedback, and virtual-reality environments for cognitive or social support (61), remain at a pilot stage but demonstrate novel opportunities to enhance accessibility and user experience. One study (61) specifically focused on populations with dementia, while another (42) explored voice recording for fall prevention. This theme corresponds primarily to Pathway 4 by fostering empowerment, emotional engagement, and social connectedness. It also contributes to Pathway 1 by improving accessibility for individuals with visual or dexterity limitations through voice-based and haptic inputs (42, 50).

### 3.3 Impact on digital health engagement and management (RQ2)

Thematic analysis of the extracted texts identified four major themes that characterize how interactive interfaces and wearable technologies influence older adults’ digital health engagement and management. These themes reflect both behavioral and experiential dimensions of engagement, encompassing usability, adherence, self-monitoring, motivational support, and broader psychosocial benefits. The themes are summarized in Table 6 and elaborated below. Each theme is interpreted through the I-WEP analytical framework.

**Table 6:** Themes identified from the thematic analysis of RQ2 (impact on digital health engagement and management).

Theme	Features/Codes	Representative studies (Ref No.)
Usability and ease of learning	Simple navigation, minimal setup, intuitive feedback; usability linked to adoption and retention	(20, 21, 24, 27, 29, 37, 38, 41, 43, 49, 50, 55, 59)
Self-monitoring and adherence	Continuous monitoring (steps, blood pressure, sleep); reinforcement of routines; improved disease management	(19, 22, 26, 28–30, 32, 40, 44–46, 51–53, 57, 59, 62)
Motivational support	Goal-setting, gamification, reminders, progress visualization; caregiver/clinician reinforcement	(26, 28–30, 32, 40, 44, 53, 59, 62). Six studies (23, 26, 28, 30, 59, 63)
Psychosocial empowerment	Increased autonomy, reduced anxiety, perceived security; improved connectedness via data sharing	(21, 22, 24, 29, 33, 34, 42, 45, 60, 61, 65)

#### 3.3.1 Theme 1: usability and ease of learning as determinants of engagement

Across 13 studies (20, 21, 24, 27, 29, 37, 38, 41, 43, 49, 50, 55, 59), older adults’ initial and sustained engagement with digital health technologies was strongly linked to perceived usability and the ease with which devices could be learned. Systems with simplified navigation, clear feedback, and minimal setup requirements were associated with higher adoption and longer wear time in 6 studies (21, 24, 29, 38, 55, 59). Conversely, 6 studies (27, 37, 41, 43, 49, 50) reported that complex interfaces and steep learning curves reduced older adults’ willingness to integrate technologies into daily routines. This theme represents Pathway 1, emphasizing that usability is the fundamental condition enabling sustained engagement and forming the foundation for subsequent behavioral adherence processes described in Pathway 2.

#### 3.3.2 Theme 2: self-monitoring and adherence to health behaviors

Wearable devices and interactive apps enabled older adults to engage in active self-monitoring in 17 studies (19, 22, 26, 28–30, 32, 40, 44–46, 51–53, 57, 59, 62), which often translated into improved adherence to physical activity, medication routines, or disease self-management. Step counters, sleep trackers, and blood pressure monitors provided immediate feedback that reinforced daily routines and promoted accountability in 10 studies (26, 28–30, 32, 40, 44, 53, 59, 62). Six studies (23, 26, 28, 30, 59, 63) reported measurable increases in physical activity or improved disease management outcomes as older adults used wearable-supported platforms consistently. This theme aligns with Pathway 2, focusing on self-monitoring and adherence. Evidence across studies shows that improvements are often significant in the short term but tend to decline after 12 to 16 weeks in 3 studies (23, 30, 59), indicating that additional motivational or social components are required for long-term sustainability.

#### 3.3.3 Theme 3: motivational support and sustained engagement

Interactive technologies served as motivational tools in 12 studies (19, 26, 29, 30, 33, 37, 38, 43–45, 59, 60). Features such as goal-setting, gamified feedback, reminders, and social comparison encouraged sustained engagement with health behaviors. Studies employing companion apps or dashboard interfaces (33, 37, 38, 43, 45) demonstrated that motivational prompts and progress visualization enhanced adherence over longer periods. In 5 studies (19, 29, 32, 45, 64), involvement of caregivers or healthcare professionals through connected platforms further reinforced motivation and accountability. This theme represents Pathway 3, which emphasizes motivational reinforcement. Evidence from 4 studies (23, 30, 44, 59) suggests that motivational effects are often temporary unless supported by human interaction, feedback personalization, or ongoing social reinforcement.

#### 3.3.4 Theme 4: psychosocial outcomes and perceived empowerment

Beyond behavioral adherence, 11 studies (21, 22, 24, 29, 33, 34, 42, 45, 60, 61, 65) reported broader psychosocial benefits associated with digital health engagement. Older adults expressed feelings of empowerment, autonomy, and security when they could monitor their own health in real time in 6 studies (22, 24, 29, 33, 34, 65). Four studies (22, 24, 33, 34) noted that wearable-supported self-monitoring reduced anxiety about chronic conditions and increased confidence in managing health. Moreover, 5 studies (32, 38, 45, 61, 64) found that technologies allowing data sharing with caregivers or clinicians promoted a sense of connectedness and reassurance. This theme corresponds to Pathway 4, which focuses on psychosocial empowerment.

### 3.4 Barriers and challenges (RQ3)

Thematic analysis of the extracted data identified four overarching themes that capture the principal barriers and challenges affecting older adults’ adoption and sustained use of interactive interfaces and wearable technologies for digital health management (Table 7). These themes concern technological usability, digital literacy, privacy and trust, as well as cost and long-term sustainability. The following synthesis distinguishes between design-related barriers, which can be mitigated through improved design or training, and structural barriers, which require broader policy or system-level solutions.

**Table 7:** Themes identified from the thematic analysis of RQ3 (barriers and challenges).

Theme	Features/Codes	Representative studies (ref no.)
Usability limitations and device discomfort	Small screens, complex menus, discomfort, connectivity problems, charging burden	(20, 22, 24, 27, 29, 37, 38, 41, 43, 49, 50, 54–56, 59)
Limited digital literacy and training needs	Difficulties in navigation, interpretation of data, troubleshooting; need for training	(19, 23, 27–29, 32, 37, 38, 41, 43, 45, 50, 54)
Privacy, trust, and data security concerns	Hesitancy to share data, fear of misuse/surveillance, lack of transparency, control concerns	(21–23, 28, 34, 42, 49, 50, 54, 60)
Cost, accessibility, and sustainability	Device expense, subscription/maintenance costs, rural/low-income disparities, obsolescence	(23, 28, 34, 36, 38, 40, 51, 53, 54, 59, 60)

#### 3.4.1 Theme 1: usability limitations and device discomfort

Usability challenges were reported as barriers in 15 studies (20, 22, 24, 27, 29, 37, 38, 41, 43, 49, 50, 54–56, 59). Older adults struggled with small screen sizes, complex menu structures, and non-intuitive interaction pathways in 9 studies (20, 24, 29, 37, 41, 43, 49, 50, 55), creating frustration and disengagement. Wearable devices also presented issues related to comfort in 8 studies (22, 27, 29, 49, 54–56, 59), such as skin irritation, bulkiness, or difficulties in fastening devices due to age-related dexterity limitations. In 6 studies (27, 37, 38, 41, 50, 56), technical glitches such as unreliable connectivity or frequent need for recharging undermined trust in the technology and discouraged long-term use. These issues represent design-contingent barriers primarily associated with Pathways 1 and 2. They can be mitigated by ergonomic improvements, simplified interaction flows, and enhanced reliability.

#### 3.4.2 Theme 2: limited digital literacy and training needs

Limited digital literacy of older adults impeded their ability to operate and maintain wearable systems or companion applications in 13 studies (19, 23, 27–29, 32, 37, 38, 41, 43, 45, 50, 54). Ten studies (19, 27, 29, 32, 38, 41, 43, 45, 50, 54) reported that many older adults required extensive guidance to interpret data outputs, navigate application settings, or troubleshoot technical problems. Without adequate training or caregiver support, engagement declined over time in 5 studies (27, 29, 32, 41, 43). Evidence from 4 studies (32, 37, 38, 43) suggests that tailored training programs and iterative learning opportunities can mitigate these challenges, but such resources are not systematically embedded into interventions. This theme relates to both Pathway 1 and Pathway 2 and demonstrates that training and scaffolding are critical enablers of technological competence. When such support is absent, these challenges evolve from design-level to structural barriers.

#### 3.4.3 Theme 3: privacy, trust, and data security concerns

Concerns about personal data privacy and security emerged as a central barrier to technology acceptance in 10 studies (21–23, 28, 34, 42, 49, 50, 54, 60). Seven studies (21, 22, 34, 42, 49, 50, 54) reported that older adults were hesitant to share health data with third parties, including healthcare providers or commercial platforms, due to fears of misuse or surveillance. Issues of trust were compounded in 5 studies (21, 22, 24, 28, 49) when devices lacked transparent explanations of data handling or when participants felt a loss of control over their personal information. This barrier is primarily structural and relates to Pathway 4, which emphasizes perceived control and trust. Evidence from 3 studies (22, 28, 49) suggests that clear consent procedures, transparent data governance, and patient-controlled data sharing can substantially reduce privacy-related resistance.

#### 3.4.4 Theme 4: cost, accessibility, and long-term sustainability

Financial cost and questions of sustainability posed additional barriers to widespread adoption in 11 studies (23, 28, 34, 36, 38, 40, 51, 53, 54, 59, 60). Commercial wearable devices were perceived as expensive in 7 studies (23, 34, 40, 51, 54, 59, 60), particularly when replacement parts, subscriptions, or continuous updates were required. In addition, 5 studies (28, 34, 36, 38, 60) noted disparities in access, with lower-income or rural-dwelling older adults less likely to maintain continuous device use. Sustainability challenges extended to device maintenance in 4 studies (36, 38, 45, 63), such as battery replacement or software obsolescence, which further discouraged long-term integration into everyday health practices. These challenges are structural in nature and require coordinated efforts in policy, financing, and infrastructure to ensure equitable access and sustainable implementation.

**Figure 2:**
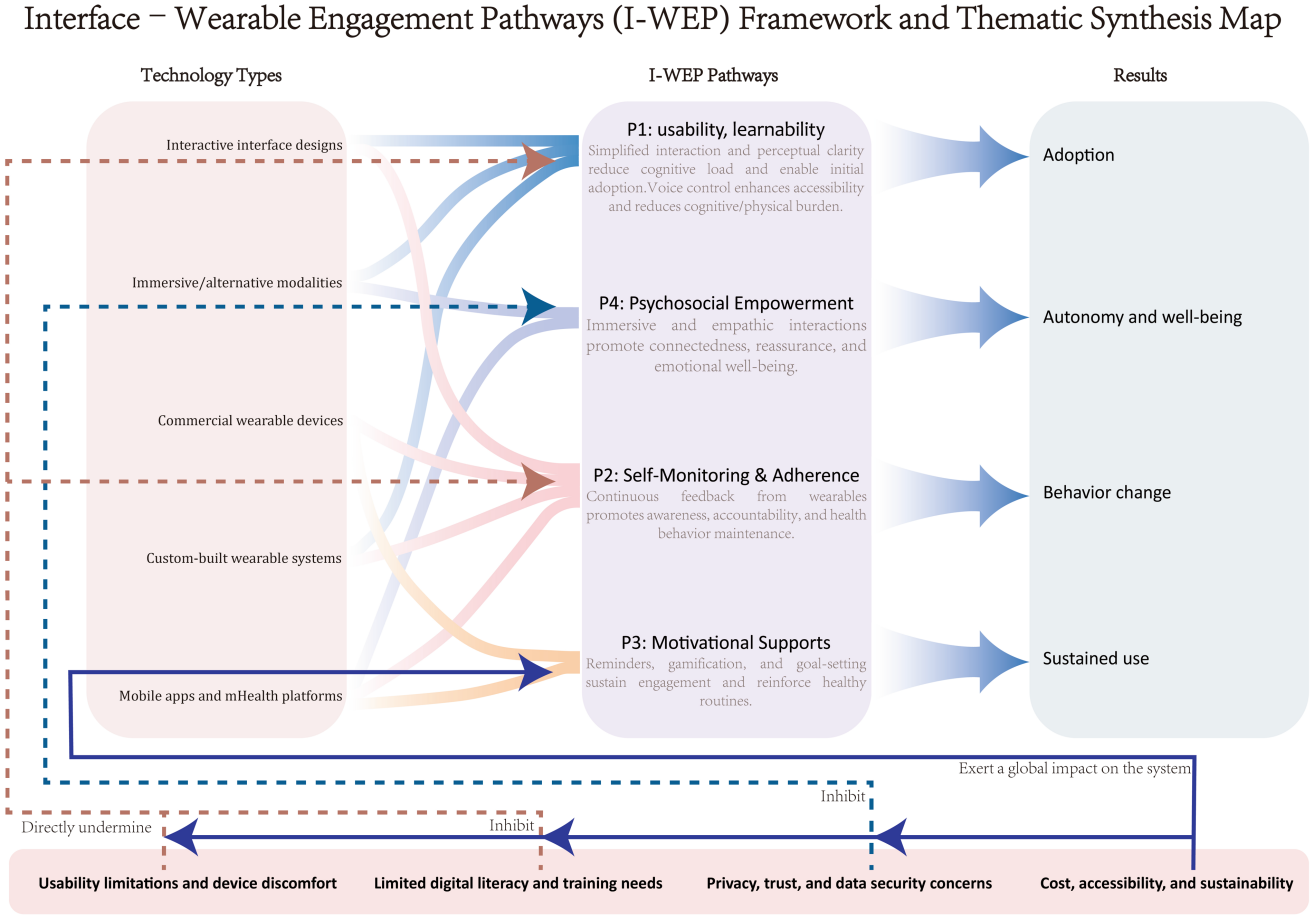
Interface-wearable engagement pathways (I-WEP) framework and thematic synthesis map.

To integrate findings across the three research questions, Figure 2 illustrates the Interface–Wearable Engagement Pathways (I-WEP) framework, which maps technology types, engagement pathways, and resulting outcomes, as well as how major barriers exert constraining influences on this system.

### 3.5 Quality assessment

All 48 included studies were appraised using the Mixed Methods Appraisal Tool (MMAT, 2018 version). The overall methodological quality varied considerably across the corpus (Table 3). Out of the 48 studies, 10 achieved a high-quality rating (scoring 4/5 or 5/5 criteria met), 25 demonstrated moderate quality (2/5 and 3/5), and 13 were assessed as low quality (0/5 and 1/5).

High-quality studies were predominantly qualitative investigations that provided detailed methodological descriptions and clear alignment between research questions, data collection, and analysis (29, 34, 43, 44, 50, 54, 61). These studies consistently met all five MMAT criteria, reflecting robust research designs and transparent reporting.

Moderate-quality studies were most common among quantitative descriptive designs. For instance, Shim et al., 2024 (39), Zhang et al., 2025 (22), Liang et al., 2025 (23), Allah et al., 2023 (37), and Villalobos et al., 2022 (45) each satisfied 3 out of 5 MMAT criteria. Such studies typically provided valid outcome measures and logical analysis but often lacked detail regarding sampling strategies, recruitment processes, or control of potential biases.

Low-quality studies were found across both descriptive and experimental categories. Several randomized or non-randomized trials, including Mathew et al., 2024 (40), Hsu et al., 2023 (20), and Wu et al., 2021 (47), reported minimal methodological detail, meeting only one or two MMAT criteria. The lowest scoring study, Lee et al., 2025 (42), failed to provide sufficient information across all five domains (0/5), making it difficult to assess methodological rigor.

When comparing by design, qualitative studies generally demonstrated stronger methodological quality compared to quantitative descriptive studies, which frequently lacked clarity on sampling procedures and handling of missing data. Mixed-methods designs showed heterogeneous results, with some (49) achieving relatively high ratings (4/5), while others (38, 47) provided limited detail and scored only 1/5.

The most common methodological weaknesses across the studies included insufficient reporting on participant recruitment, lack of transparency in addressing non-response or attrition, and limited detail on integration of qualitative and quantitative strands in mixed-methods designs. Conversely, strengths observed in higher-quality studies were comprehensive reporting of analytic processes, alignment of design and objectives, and provision of rich contextual detail.

In summary, the MMAT assessment highlights notable variability in methodological rigor. While several studies (29, 34, 43, 44, 50, 54, 61) exemplify high-quality standards, a substantial proportion of the literature is constrained by limited methodological transparency. These findings emphasize the need for more rigorous reporting and design practices in future research on interactive interfaces and wearable technologies for older adults.

## 4 Discussion

This systematic review synthesized evidence from 48 studies on interactive interfaces and wearable technologies for older adults, addressing three research questions related to technology types, their impact on health engagement, and barriers to adoption. The findings demonstrate both the diversity of technological innovations in this field and the complex set of factors shaping older adults’ engagement and sustained use. Importantly, this review approached interactive interfaces and wearable technologies not as parallel categories but as interdependent components of a unified socio-technical system. This integration reflects how data sensing and user interaction jointly construct health engagement processes, bridging the gap between hardware functionality and user experience.

The thematic synthesis for RQ1 revealed five overarching categories of technologies: commercial wearable devices, custom-built systems, mobile health platforms, user-centered interface designs, and immersive or alternative modalities. Across studies, three analytical patterns emerged. First, there is a persistent tension between technological sophistication and everyday usability: systems that maximize sensing precision often impose cognitive or physical burdens that undermine daily engagement. Second, studies that integrated interfaces and wearables within a single feedback loop consistently demonstrated stronger adherence effects than those examining devices or interfaces in isolation, underscoring the interdependence of design layers within the I-WEP framework. Third, while commercial technologies dominated the evidence base, bespoke and inclusive interface designs were more effective in addressing sensory and cognitive diversity, revealing a research gap in adaptive, age-tailored multimodal systems. These cross-study insights shift the interpretation from typological description toward a structural understanding of how interface–wearable systems co-evolve to balance precision, usability, and inclusivity.

For RQ2, four themes emerged regarding the impact on health engagement and management: usability and ease of learning, self-monitoring and adherence, motivational support, and psychosocial empowerment. Across the reviewed studies, several conceptual patterns and tensions became apparent. First, engagement trajectories tended to follow a “rise–plateau–decline” curve, suggesting that initial novelty and motivational features generate short-term adherence that is difficult to sustain without evolving feedback or human reinforcement. Second, there exists a design trade-off between simplicity and functionality: interfaces optimized for ease of use sometimes limited behavioral depth, whereas complex systems risked cognitive overload. Third, psychosocial empowerment consistently outlasted behavioral adherence, implying that emotional and relational factors may constitute a more stable foundation for long-term digital health engagement. These observations emphasize that engagement is not a static outcome but a dynamic process shaped by iterative interaction between usability, motivation, and social reinforcement within the I-WEP pathways. These findings collectively illustrate that interface design and wearable sensing are not separate contributors but interact dynamically: interface feedback transforms sensor data into comprehension and motivation, while wearable accuracy and comfort influence the perceived usefulness of interface outputs.

RQ3 identified several barriers that constrain adoption and sustained use, including usability challenges, limited digital literacy, concerns about privacy and data security, and financial cost and sustainability issues. When viewed analytically across studies, three overarching tensions emerge. First, many usability and literacy barriers stem not from user deficits but from design misalignments: technologies implicitly assume younger-user competencies, producing a structural “design–user gap.” Second, despite recurring emphasis on privacy and cost, few interventions addressed these issues through co-design or participatory strategies, revealing a disconnect between user concerns and system design priorities. Third, evidence across contexts suggests that digital engagement is constrained by a cumulative interaction of individual (skills), technical (usability), and systemic (policy) barriers, rather than any single obstacle. These interlocking constraints highlight that sustainable adoption requires multilevel interventions aligning design, training, and governance rather than isolated technical fixes. Together, these findings illustrate that engagement is shaped by both design-level features and broader structural factors.

In interpreting these findings, it is also essential to consider how the characteristics of the included studies shape the strength, direction, and generalizability of the available evidence. First, the geographic concentration of studies in high-income or rapidly developing regions, particularly East Asia, North America, and parts of Europe, suggests that much of the current evidence reflects contexts with strong digital infrastructure, higher baseline technology adoption, and greater institutional support for digital health. This geographic skew raises concerns regarding global applicability, as older adults in low-income settings may face distinct barriers related to affordability, connectivity, and health-system capacity. Second, the predominance of non-randomized, feasibility, and pilot study designs limits the ability to draw causal inferences about effectiveness. While these designs provide important insights into usability, acceptability, and early feasibility, they offer weaker evidence regarding long-term health outcomes or sustained behavioral change. Only a small subset of randomized controlled trials contributes high-certainty evidence, narrowing the strength of conclusions that can be drawn about comparative effectiveness across devices or interface types. Third, although caregivers play crucial roles in supporting digital health behaviors, only 13 of the 48 studies explicitly involved caregivers, and most interventions were designed primarily for individual users. This gap suggests that the current evidence base may underestimate the relational and interdependent nature of aging and technology use. Collectively, these study-level characteristics reveal that the existing literature provides a valuable but partial picture. This picture reflects certain socioeconomic contexts, emphasizes early-stage feasibility, and fails to fully incorporate the caregiving ecosystem that is central to older adults’ digital health engagement and management.

These findings resonate with prior reviews of digital health technologies for older adults, which have similarly emphasized usability and digital literacy as key determinants of engagement. For instance, Wang et al. found that satisfaction and learnability were among the most frequently evaluated usability dimensions and critical for app acceptance among older adults (66). Other authors demonstrated that design features such as simple navigation and persuasive elements such as reminders, goal setting, enhance adoption and health-related outcomes (12). Moore et al. also highlighted that motivation, device purpose alignment with user expectations, and ease of use were major influences on long-term adherence (11). A recent review article (67) underscores the importance of age-friendly interface features like enlarged text, voice interactions, and error-tolerant design. Additionally, Takano et al. reported that standardized usability scales and user perception of usefulness and ease are strongly associated with positive UX (User Experience) and continued usage (68).

However, unlike prior reviews that treated user interface design and wearable technologies as separate domains, our synthesis explicitly examined how these two interact as a coupled system within digital health engagement. This integrative lens, informed by the Interface–Wearable Engagement Pathways (I-WEP) framework, elucidates the behavioral and experiential mechanisms linking sensor-based monitoring, interface feedback, and user motivation. It further clarifies why older adults’ sustained engagement depends not only on hardware ergonomics or software usability alone but on their iterative interaction. The current synthesis demonstrates how these domains intersect, underscoring that wearable hardware must be supported by user-centered software and that psychosocial outcomes are as critical as physiological metrics. Furthermore, the identification of immersive modalities such as VR and voice interfaces highlights an emerging area not yet extensively covered in earlier reviews.

The findings carry several practical implications. For designers, the evidence underscores the necessity of age-sensitive interface adaptations—larger icons, simplified navigation, high-contrast visuals, and multimodal feedback are not optional but essential features (21, 24, 47). For healthcare providers, wearable devices and companion apps should be viewed not merely as monitoring tools but as enablers of self-management and psychosocial empowerment (22, 42). Integrating caregiver and clinician support into platforms may improve adherence and motivation (38, 45). For policymakers, cost and equity considerations demand attention. Subsidizing devices for low-income populations or incorporating wearables into publicly funded health programs may help reduce disparities in access (36, 40, 63). Transparent data governance frameworks are also necessary to address privacy and trust concerns (21, 60).

Beyond these practical implications, a broader innovation agenda emerges from the synthesis. Future development should move beyond optimizing existing usability parameters to reimagining adaptive, context-aware systems capable of evolving with users’ functional changes and motivational states. Integrating artificial intelligence (AI)–driven personalization, emotion-sensitive feedback, and multimodal interaction (voice, touch, gesture) represents an urgent but under-explored direction for both academia and industry. Similarly, participatory co-design with older adults and caregivers is crucial for aligning technical innovation with lived experience, ensuring that new systems address real-world diversity rather than idealized user profiles. At the policy level, coordinated public–private partnerships could accelerate translation from prototypes to sustainable deployment, accompanied by data-governance standards that balance accessibility, privacy, and interoperability.

This review followed PRISMA guidelines and applied thematic analysis to synthesize evidence across diverse study designs. A strength lies in its dual focus on both interactive interface design and wearable technologies, offering a more holistic understanding of digital health engagement among older adults. However, several limitations must be acknowledged. First, the methodological quality of included studies was variable: only 10 studies achieved high MMAT scores, while many were of moderate or low quality, often due to poor reporting of sampling or bias control (31, 39, 54, 57, 64). Second, heterogeneity in interventions and outcomes limited the comparability of results and precluded meta-analysis. Third, although multiple databases were searched, language restrictions and the five-year publication window may have excluded relevant studies. Finally, thematic analysis relies on interpretation and may be subject to reviewer bias, although this was mitigated by independent coding and consensus procedures.

Looking forward, advancing this field requires concurrent progress on three fronts. Methodologically, studies should adopt longitudinal, mixed-methods, and multi-context designs that capture sustained engagement dynamics rather than short-term usability outcomes. In terms of human-centered design, future work should emphasize adaptive and emotion-responsive interfaces that foster long-term trust, motivation, and empowerment. At the systems and policy level, scalable infrastructures—interoperable data standards, equitable cost models, and training frameworks—are essential to ensure inclusion across socioeconomic contexts. Building on these directions, future studies should also prioritize methodological rigor and transparency, ensuring clear reporting of recruitment, attrition, and bias management. More randomized controlled trials with larger, diverse samples are needed to assess effectiveness beyond feasibility. In addition, future work should explore long-term engagement and sustainability, addressing issues of device maintenance, software updates, and cost. Research on immersive modalities such as VR and voice-based systems should be expanded to determine their potential for accessibility. Ultimately, the path forward depends on bridging methodological rigor, inclusive design, and supportive policy ecosystems, transforming digital health technologies from experimental tools into equitable instruments for healthy and dignified aging.

## 5 Conclusion

This systematic review synthesized evidence from 48 empirical studies to clarify current knowledge regarding how interactive interfaces and wearable technologies support older adults’ digital health engagement and management. Across diverse technological modalities and study designs, the evidence collectively demonstrates that such technologies can meaningfully enhance older adults’ ability to monitor their health, adhere to recommended behaviors, and feel more confident and supported in daily health management. However, this potential is inconsistently realized. Three overarching conclusions emerge.

First, the effectiveness of wearable–interface systems depends primarily on usability, integration, and support rather than on the technical sophistication of sensors. Older adults benefit most when technologies are simple to operate, provide clear and actionable feedback, and are embedded within supportive ecosystems that may include training, caregiver involvement, and clinician communication channels.

Second, despite growing research activity, substantial structural and individual barriers continue to constrain equitable engagement. Usability limitations, digital literacy gaps, concerns about privacy and data governance, and the financial burden of devices collectively restrict sustained adoption. These barriers indicate that technological solutions alone are insufficient; successful implementation requires attention to accessibility, trust, and sustained support.

Third, the current evidence base remains methodologically heterogeneous, with many studies relying on small samples, short follow-up periods, and limited outcome standardization. This limits the ability to draw firm conclusions about long-term effectiveness or to compare results across interventions.

Taken together, these findings highlight a clear agenda for future work: (1) design technologies grounded in age-centered Human-Computer Interaction (HCI) principles; (2) integrate training and caregiver/clinician support as core components rather than optional additions; (3) address equity and cost barriers through policy and system-level interventions; and (4) strengthen the evidence base through rigorous, longer-term, and comparative studies with standardized engagement outcomes.

In conclusion, interactive interfaces and wearable technologies offer meaningful opportunities to enhance older adults’ digital health management, but realizing this promise requires coordinated progress in design, implementation, and policy to ensure that these technologies are accessible, trustworthy, and sustainable for all older adults.

## Data Availability

The original contributions presented in the study are included in the article/supplementary material, further inquiries can be directed to the corresponding authors.
